# 

**Published:** 1892-05

**Authors:** 


SUBSCRIBE FOR THE

### International Dental Journal,

THE ORGAN OF THE PROFESSION.

**Published by the International Dental Publication Company.**

LOUIS JACK, President, Philadelphia.
BENJAMIN LORD, Vice-President, New York.
C. N. PEIRCE, Treasurer, Philadelphia.
GEO. S. ALLEN, Secretary, 51 West Thirty-seventh St., New York.

**Publication Office, 716 Filbert St., Philadelphia, Pa.**

STOCKHOLDERS.

| | |
|---|---|
| Frank Abbott | New York, N.Y. |
| Geo. S. Allen | New York, N.Y. |
| W. W. Allport | Chicago, Ill. |
| R. R. Andrews | Cambridge, Mass. |
| H. A. Baker | Boston, Mass. |
| A. E. Baldwin | Chicago, Ill. |
| W. C. Barrett | Buffalo, N.Y. |
| A. P. Beale | Philadelphia, Pa. |
| S. T. Beale, Jr. | Philadelphia, Pa. |
| C. S. Beck | Wilkesbarre, Pa. |
| G. V. Black | Jacksonville, Ill. |
| C. F. W. Bodecker | New York, N.Y. |
| E. A. Bogue | New York, N.Y. |
| W. G. A. Bonwill | Philadelphia, Pa. |
| C. A. Brackett | Newport, R.I. |
| Thomas Bradley | New York, N.Y. |
| Edward C. Briggs | Boston, Mass. |
| Albert H. Brockway | Brooklyn, N.Y. |
| A. E. Brown | Chicago, Ill. |
| Wm. Carr | New York, N.Y. |
| Geo. H. Chance | Portland, Oregon. |
| G. F. Cheney | St. Johnsbury, Vt. |
| Dwight M. Clapp | Boston, Mass. |
| John T. Codman | Boston, Mass. |
| Chas. D. Cook | Brooklyn, N.Y. |
| J. N. Crouse | Chicago, Ill. |
| Edw. T. Darby | Philadelphia, Pa. |
| H. S. Draper | Boston, Mass. |
| Wm. H. Dwinelle | New York, N.Y. |
| A. R. Eaton | Elizabeth, N.J. |
| Chas. J. Essig | Philadelphia, Pa. |
| J. N. Farrer | New York, N.Y. |
| T. Fillebrown | Portland, Me. |
| W. B. Finney | Baltimore, Md. |
| T. A. Fletcher | New York, N.Y. |
| M. W. Foster | Baltimore, Md. |
| Chas. E. Francis | New York, N.Y. |
| E. C. Fundenberg | Pittsburg, Pa. |
| W. F. Fundenberg | Pittsburg, Pa. |
| W. H. Fundenberg | Pittsburg, Pa. |
| E. S. Gaylord | New Haven, Conn. |
| J. P. Geran | Brooklyn, N.Y. |
| A. H. Gilson | Boston, Mass. |
| S. H. Guilford | Philadelphia, Pa. |
| John I. Hart | New York, N.Y. |
| O. E. Hill | Brooklyn, N.Y. |
| J. F. P. Hodson | New York, N.Y. |
| J. Morgan Howe | New York, N.Y. |
| Robert Huey | Philadelphia, Pa. |
| A. O. Hunt | Iowa City, Iowa. |
| Louis Jack | Philadelphia, Pa. |
| V. H. Jackson | New York, N.Y. |
| C. R. Jefferis | Wilmington, Del. |
| C. A. Kingsbury | Philadelphia. Pa. |
| Edw. C. Kirk | Philadelphia, Pa. |
| C. R. E. Koch | Chicago, Ill. |
| W. T. La Roche | Harrington Park, N.J. |
| W. K. Leech | Philadelphia, Pa. |
| F. A. Levy | Orange, N.J. |
| Wilbur F. Litch | Philadelphia, Pa. |
| J. Bond Littig | New York, N.Y. |
| Benjamin Lord | New York, N.Y. |
| Geo. W. Lovejoy | Montreal, Ont. |
| John S. Marshall | Chicago, Ill. |
| H. J. McKellops | St. Louis, Mo. |
| Charles McManus | Hartford, Conn. |
| James McManus | Hartford, Conn. |
| Dan'l Neal McQuillan | Philadelphia, Pa. |
| Horatio C. Meriam | Salem, Mass. |
| W. D. Miller | Berlin, Germany. |
| W. E. Page | Boston, Mass. |
| S. B. Palmer | Syracuse, N.Y. |
| C. B. Parker | Brooklyn, N.Y. |
| D. D. Peabody | Stoneham, Mass. |
| C. N. Peirce | Philadelphia, Pa. |
| S. G. Perry | New York, N.Y. |
| W. A. Phreaner | Philadelphia, Pa. |
| Chas. E. Pike | Philadelphia, Pa. |
| W. E. Pinkham | New York, N.Y. |
| W. H. Potter | Boston, Mass. |
| Chas. P. Pruyn | Chicago, Ill. |
| H. C. Register | Philadelphia, Pa. |
| Z. T. Sailer | New York, N.Y. |
| L. D. Shepard | Boston, Mass. |
| Geo. R. Shidle | Pittsburg, Pa. |
| Eugene H. Smith | Boston, Mass. |
| B. Holly Smith | Baltimore, Md. |
| H. A. Smith | Cincinnati, Ohio. |
| S. G. Stevens | Boston, Mass. |
| W. X. Sudduth | Minneapolis, Minn. |
| Eugene S. Talbott | Chicago, Ill. |
| Ambler Tees | Philadelphia, Pa. |
| J. D. Thomas | Philadelphia, Pa. |
| James Truman | Philadelphia, Pa. |
| Wm. H. Trueman | Philadelphia, Pa. |
| W. W. Walker | New York, N.Y. |
| I. F. Wardwell | Stanford, Conn. |
| G. W. Warren | Philadelphia. Pa. |
| T. S. Waters | Baltimore, Md. |
| Geo. W. Weld | New York, N.Y. |
| Julius G. W. Werner | Boston, Mass. |
| Jacob L. Williams | Boston, Mass. |
| R. B. Winder | Baltimore, Md. |
| C. A. Woodward | New York, N.Y. |
| J. A. Woodward | Philadelphia, Pa. |
| W. A. Woodward | New York, N.Y. |
| W. J. Younger | San Francisco, Cal. |

The officers of the International Dental Publication Company serve without salary, the investments of the stockholders merely guarantee the success of the enterprise, ALL PROFITS being devoted to the enlargement or improvement of the International Dental Journal.

The support of every dentist having the welfare of his profession at heart is earnestly solicited. Subscription, $2.50 per Annum.

*THE INTERNATIONAL DENTAL PUBLICATION CO.*

### IMPORTANT NOTICE.

For the convenience of subscribers to the INTERNATIONAL DENTAL JOURNAL, who often find it difficult to remit small amounts through the mail, we have established agencies in different localities (in most cases with dealers in Dental Goods, with whom dentists have frequent business transactions), WHERE EITHER NEW OR RENEWAL OF SUBSCRIPTIONS MAY BE PAID.

### International Dental Publication Co.,

PUBLISHERS.

### LIST OF AGENTS.

| NAME. | ADDRESS. | BUSINESS. |
| --- | --- | --- |
| Fred. W. Smith | Binghamton, N. Y | Dental Depot. |
| F. M. Allen, D.D.S | Birmingham, Ala | Dental Depot and Dentist. |
| Buffalo Dental Mfg. Co | 587 and 589 Main Street, Buffalo, N. Y | Dental Depot. |
| Boston Dental Mfg. Co | 174 Tremont Street, Boston | Dental Supplies. |
| B. L. Knapp & Co | 161 Tremont St., Boston, Mass | Dentists' Materials. |
| Illinois Dental Mfg. Co | 85 Fifth Ave., Chicago, Ill | Dental Supplies. |
| S. A. Crocker & Co | 119 W. Fifth St., Cincinnati, Ohio | Ohio Dental and Surg. Depot. |
| George W. Fels | 139 W. Sixth Street, Cincinnati, Ohio | Dental Depot. |
| M. A. Spencer & Co. | 134 W. Seventh Street, Cincinnati, O. | Surg. and Dent. Instruments. |
| J. Durbin | Denver, Col | Dental Goods. |
| Marshall Dental Mfg. Co | 309 Seventh Street, Des Moines, Iowa. | |
| Keller Dental Co | Fort Wayne, Ind. | Dental Manufacturers. |
| C. B. Woodward & Co | Fort Wayne, Ind. | Dental Depot. |
| E. S. Holmes | Grand Rapids, Mich | Dentist. |
| W. C. Messinger | Hartford, Conn | Dental Supplies. |
| J. E. Bodine & Co | Indianapolis, Ind. | Dental Depot. |
| Dr. A. W. Clark | Jefferson, N. Y | Dental Supplies. |
| R. I. Pearson & Co | 906 Grand Avenue, Kansas City, Mo | Surgical and Dental Depot. |
| T. P. Wagoner | 64 Main Street, Knightstown, Ind | Dental Goods. |

| NAME. | ADDRESS. | BUSINESS. |
|---|---|---|
| L. S. Kellner | Knoxville, Tenn | East Tennesee Dental Depot. |
| Wells Dental Depot | Corner Spring and First Streets, Los Angeles, Cal. | |
| L. J. Frazel | 438 W. Walnut Street, Louisville, Ky. | Louisville Dental Depot. |
| E. G. Fowler | Montgomery, Ala | Dental Depot. |
| Morrison Bros | Nashville, Tenn | Dental Depot. |
| Osman & Cook | Newark, N. J., Box 265 | Newark Dental Depot. |
| E. L. Washburn & Co | New Haven, Conn | Dental Depot. |
| Stuart & Adams | New Orleans, La | Dental Depot. |
| C. Ash & Sons | 30 East 14th St., New York | Dental Goods. |
| E. Steiger & Co. | 32 Park Place, New York. | |
| Dr. C. E. Fitzgerald | Paris, Tenn | Dentist. |
| J. B. Dunlevy | Pittsburgh, Pa | Dental Depot. |
| Lee S. Smith | 52 6th St., Pittsburgh, Pa | Dental Depot. |
| George C. Frye | Portland, Maine | Dental Depot. |
| Dr. F. W. Seabury | 294 Westminster Street, Providence, R. I. | Dentist. |
| Charles Oehlmann | Quincy, Ill. | Dental Depot. |
| A. Grant | 917 Main St., Richmond, Va. | Dental Depot. |
| James W. Edwards, | 5½ Kearny Street, San Francisco, Cal. | |
| J. H. A. Folkers & Bro. | 815 Market Street, San Francisco, Cal. | |
| Wm. M. Williams | Springfield, Mass | Dental Depot. |
| John Rowan | St. Louis, Mo | Dental Depot. |
| St. Louis Dental Mfg. Co | St. Louis, Mo | Dental Depot. |
| Minnesota Dental Mfg. Co | St. Paul, Minn. | |
| M. F. Patterson | St. Paul, Minn | Dental Goods. |
| H. C. Leyden | Syracuse, N. Y. | Dental Depot. |
| The Buckhart Dental Supply Co | 930 Pacific Avenue, Tacoma, Wash | Dental Goods. |
| J. P. Stansell | Temple, Texas | Dental Depot. |
| Ransom & Randolph | Toledo, Ohio | Dental Depot. |
| S. B. Chandler Dent. Mfg. Co. | Toronto, Canada | Dental Goods. |
| C. H. Hubbard | 44 Adelaide Street W., Toronto, Canada. | |
| E. J. Lewis | 1215 Pennsylvania Ave., Washington, D.C. | Dealers in Dental Materials. |
| Robinson & Armstrong | Watertown, N. Y | Dental Supplies. |

